# Measuring impact of systematic reviews using individual participant data: evidence from clinical guidelines

**DOI:** 10.1186/1745-6215-14-S1-P123

**Published:** 2013-11-29

**Authors:** Claire Vale, Larysa Rydzewska, Lesley Stewart, Maroeska Rovers, Jonathan Emberson, François Gueyffier

**Affiliations:** 1 MRC Clinical Trials Unit Hub for Trials Methodology Research, London, UK; 2 Centre for Research and Dissemination, University of York, York, UK; 3 The Radboud University Nijmegen Medical Centre, Nijmegen, The Netherlands; 4 UMR5558, CNRC, University of Lyon, Lyon, France; 5 Clinical Trial Service Unit and Epidemiological Studies Unit, Oxford, UK

## Background

Systematic reviews (SRs) using individual participant data (IPD) are often referred to as the gold standard due to improved precision and reliability. Furthermore, they may identify treatment by participant subgroup interactions, enabling better targeting of treatments. IPD SRs have enormous potential to influence clinical guidelines, however their uptake by guideline developers, as Level 1 evidence, is unclear.

## Objectives

We aimed to assess the impact of IPD SRs of interventions on clinical guidelines across a variety of healthcare areas.

## Methods

30 eligible IPD SRs published from 2008 to 2010 spanning clinical areas including cancer, cardiovascular disease, and epilepsy were identified from records of the Cochrane IPD Meta-analysis Methods Group. Clinical guidelines across corresponding healthcare areas, published or revised since 2010, were searched for references to these IPD SRs. Details of recommendations based on the results of these reviews and citations of other relevant SRs were collected.

## Results

Searches identified 227 potentially relevant clinical guidelines. Preliminary results are based on four IPD SRs in cancer and 34 relevant guidelines. Each of the four IPD SRs was cited in 2 to 5 guidelines, however citations were only identified in 11/34 guidelines (32%). Three of the 14 guidelines also cited aggregate data SRs, with little distinction between results of IPD or aggregate data SRs.

Further results for all 30 IPD SRs and 227 guidelines will be presented.

## Conclusions

Results of this study will help develop guidance regarding how best to use IPD SRs to inform guidelines and thus impact on clinical practice.

